# The complete chloroplast genome of *Plantago fengdouensis* (Plantaginaceae): an endemic and Endangered species from China

**DOI:** 10.1080/23802359.2019.1694851

**Published:** 2019-12-09

**Authors:** Qian Wang, Li-Hui Mao, Bo Ding, Man-Ting Li, Zhi-Xi Fu, Hong-Ping Deng

**Affiliations:** a School of Life Sciences, Chongqing Key Laboratory of Plant Resource Conservation and Germplasm Innovation, Institute of Resources Botany, Southwest University, Chongqing, P. R. China;; b Research and Development Center of Flower, Zhejiang Academy of Agricultural Sciences, Zhejiang, P. R. China;; c Biotechnology Research Center, Southwest University, Chongqing, P. R. China;; d College of Life Sciences, Sichuan Normal University, Chengdu, P. R. China

**Keywords:** *Plantago fengdouensis*, chloroplast genome, phylogenomics

## Abstract

This study was the first report about the complete chloroplast genome of *Plantago fengdouensis* (Plantaginaceae). The circular whole cp genome of *P. fengdouensis* was in a total length 164,976 bp with the typical quadripartite structure of angiosperms, containing two inverted repeats (IRs) of 38,644 bp separated by a large single-copy (LSC) region and a small single-copy (SSC) region of 82,972 and 4716 bp, respectively. The plastid genome of *P. fengdouensis* contains 113 genes, including 79 protein-coding genes, 4 ribosomal RNA genes, and 30 transfer RNA genes. The overall GC content of *P. fengdouensis* plastid genome is 38.0% and the corresponding values in LSC, SSC, and IR regions are 36.6, 30.2, and39.9%, respectively.

*Plantago* L. (Plantaginaceae) is a cosmopolitan genus with about 250 species, 22 species in China (Rahn 1996; Hassemer et al. 2015). *Plantago fengdouensis* (Z. E. Zhao & Yong Wang) Yong Wang & Z. Yu Li is an amphibious plant with a highly restricted distribution, occurring only between 140 and 160 m on the three alluvial islets in Fengdu and Zhongxian counties and Banan district within the Three Gorges Dam area. The natural habitats were permanently submerged by the water project in June 2003 and the living plants are conserved and reintroduced in recent years. It has ecological value in western China. So far, the complete chloroplast genome of *Plantago fengdouensis* has not yet been published.

Fresh leaves from one individual *Plantago fengdouensis* were collected from Jiangjin by Man-Ting Li, and the voucher specimen (JJS2019301) was deposited in the herbarium of Southwest University (former herbarium of Southwest Normal University, SWCTU). The total genomic DNA was extracted using the modified CTAB method (Doyle and Doyle 1987). The genome skimming sequencing was conducted, with 150 bp paired-end (PE) reads on the Illumina HiSeq 2000 platform. Total 6.1 Gb data were generated. The raw reads were filtered for low-quality bases (PHRED <20) by NGSQC Toolkit v2.3.3 (Patel and Jain 2012).The clean reads were then used to assemble the complete plastid genome using the plastid genomes *P. depressa* (NC 041161) as reference. We performed the assembly and annotation using Geneious 10.1.3 and adjusted the genes manually to make sure that were maintained as open reading frames.IR boundaries for the draft plastome were confirmed by BLAST. Finally, we obtained a chloroplast genome of *P. fengdouensis* and submitted the whole genome to GenBank (accession number: MN418388).

The plastome of *P. fengdouensis* was found to possess a total length 164,976 bp with the typical quadripartite structure of angiosperms, containing two inverted repeats (IRs) of 38,644 bp separated by a large single-copy (LSC) region and a small single-copy (SSC) region of 82,972 and 4716 bp, respectively. The plastid genome of *P. fengdouensis* contains 113 genes, including 79 protein-coding genes, 4 ribosomal RNA genes, and 30 transfer RNA genes. The overall GC content of *P. fengdouensis* plastid genome is 38.0% and the corresponding values in LSC, SSC, and IR regions are 36.6, 30.2, and 39.9%, respectively.

We also used the coding sequences of *P. fengdouensis* and another 10 plastomes to reconstruct a maximum likelihood tree through RAxML (Stamatakis 2014) under the GTRGAMMA substitution model, with 1000 bootstraps on CIPRES website (Miller et al. 2010). Phylogenetic analysis showed that *P. fengdouensis* was closely related to *P. depressa* within the genus *Plantago* with strong support (Figure 1). The complete plastome sequence of *P. fengdouensis* will provide a useful resource for the conservation genetics of this species as well as for the phylogenetic studies for Plantaginaceae.

**Figure 1.**
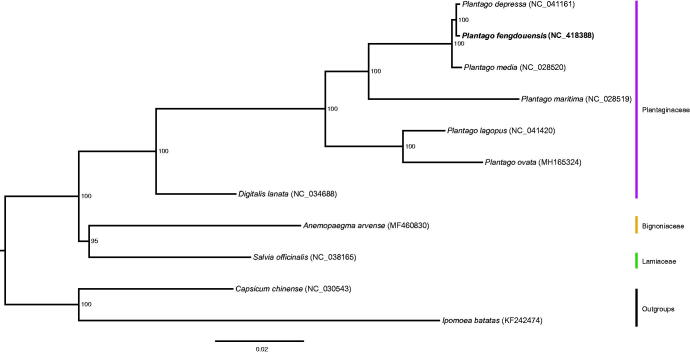
Phylogenetic tree based on the coding sequences of 11 plastomes. Bootstrap support value from 1000 replicates is shown above branches. All the plastome sequences are available in GenBank, with the accession numbers listed right to their scientific names. The new plastome obtained in this study is shown with bold.
